# Aggressive B-cell Lymphoma Masquerading as Benign Epstein-Barr Virus (EBV)-Related Splenomegaly: An Analysis of Diagnostic Anchoring Bias

**DOI:** 10.7759/cureus.110931

**Published:** 2026-06-15

**Authors:** Amrut Savadkar, Ipsita Chauhan, Mansi Sharma, Suhasini Rallabandi, Pratibha Patil, Vaishali Benaka Hebbar, Rahul Kashyap

**Affiliations:** 1 Internal Medicine, St. Joseph's Medical Center, Stockton, USA; 2 Obstetrics and Gynaecology, Flushing Hospital Medical Center, Flushing, USA; 3 Internal Medicine, UNC Health Wayne, Goldsboro, USA; 4 Internal Medicine, Banner Baywood Medical Center, Phoenix, USA; 5 Pediatrics, Community Medical Centers, Tracy, USA; 6 Pediatrics, Community Medical Centers, Livermore, USA; 7 Medicine, Drexel University College of Medicine, Philadelphia, USA; 8 Research, Global Remote Research Scholars Program, Princeton Junction, USA; 9 Critical Care Medicine, Mayo Clinic, Rochester, USA; 10 Research, WellSpan Health, York, USA

**Keywords:** b cell lymphoma, diagnostic anchoring, diagnostic challenges, ebstein-barr virus, splenomegaly

## Abstract

We report a case involving a 52-year-old male who was initially diagnosed with Epstein-Barr virus (EBV)-related splenomegaly and subsequently identified as having an aggressive B-cell lymphoproliferative disorder. Despite multiple initial diagnostic tests yielding negative results, persistent clinical suspicion due to the worsening patient's condition warranted further investigation, ultimately establishing the correct diagnosis. This case underscores the diagnostic challenges in distinguishing benign viral-associated splenomegaly from underlying malignant lymphoproliferative disorders and highlights the importance of maintaining clinical vigilance when initial diagnostic findings are discordant with the clinical presentation.

## Introduction

Splenomegaly poses a diagnostic challenge. It is a nonspecific clinical finding with wide differential diagnoses, encompassing conditions from benign infectious causes to aggressive hematologic malignancies. Epstein-Barr virus (EBV) infection can cause reactive lymphoid hyperplasia, resulting in splenomegaly [1]. Additionally, it is associated with various lymphoproliferative disorders, including diffuse large B-cell lymphoma (DLBCL) and EBV-positive lymphomas [2,3]. Distinguishing between these entities is critical, as their management and prognosis differ substantially [4,5]. EBV serology may indicate prior or current infection but cannot distinguish a benign reactive process from a more serious malignant disease [6]. Similarly, an excisional lymph node biopsy has more than 98% sensitivity, but it may yield false-negative results [7]. We present a case in which initial negative lymph node biopsy results and positive EBV serology led to diagnostic anchoring, delaying the diagnosis of aggressive stage IV B cell lymphoma. This, in turn, led to a delay in the initiation of appropriate chemotherapy, which is crucial for improving the prognosis of lymphoma. To avoid such diagnostic anchoring, one must continuously re-evaluate differential diagnoses, especially in light of new clinical information. Being aware of the diagnostic limitations of tests and being vigilant about other, more serious diseases ensures timely diagnosis and treatment, thereby improving patient outcomes.

## Case presentation

A 52-year-old male with a history of obstructive sleep apnea presented to his primary care physician with abdominal pain for two days. Pain was sharp, located mainly in the left upper quadrant with no radiation and no aggravating or relieving factors. The patient also reported a two-week history of subjective fevers with a non-productive cough. His physical examination was benign, except for a palpable spleen. He was given symptomatic management and was referred to have an abdominal ultrasound. However, he was unable to schedule the ultrasound. Progressive abdominal pain prompted two emergency department (ED) visits within the following week, where CT imaging of the abdomen revealed 19 cm splenomegaly with internal hypo-enhancement of unclear etiology (Figure 1), raising concern for splenic infarct or laceration. However, CT angiography of the abdomen did not demonstrate evidence of splenic artery thrombosis. The patient did not have any history of trauma. He was referred for outpatient hematology evaluation, but he was unable to be seen prior to his subsequent ED visit.

**Figure 1:**
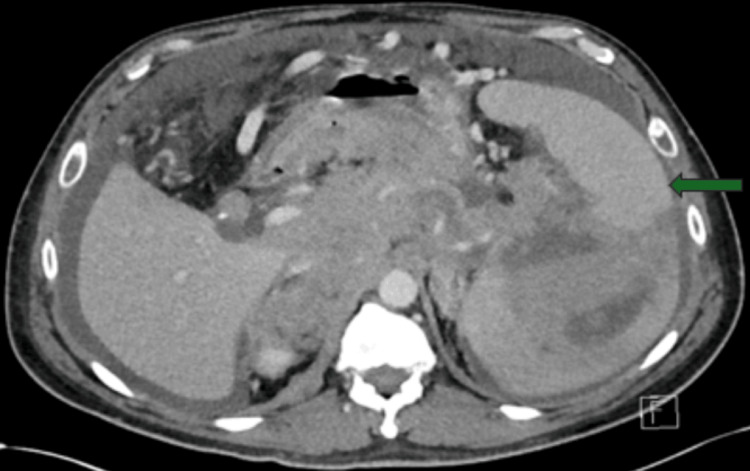
Cross-sectional CT abdomen showing 19 cm splenomegaly The green arrow indicates an enlarged spleen.

Approximately three weeks later, he returned to the ED for worsening abdominal pain. A repeat CT of the abdomen demonstrated persistent splenomegaly and newly identified mild retroperitoneal lymphadenopathy. Given the worsening abdominal pain and development of lymphadenopathy, the patient was admitted for a more comprehensive diagnostic workup with oncology. This included hypercoagulable evaluation (to exclude splenic infarct), peripheral blood flow cytometry, bone marrow biopsy, and serologic testing for HIV and EBV. HIV antibody screen and QuantiFERON tests were negative. The bone marrow biopsy revealed no evidence of malignancy, and flow cytometry results did not show any abnormal immunophenotypic cancerous cells. However, positive EBV serology (Table 1) led to a provisional diagnosis of EBV-associated splenomegaly, and the patient was discharged home with analgesics, and he was advised to follow up with his primary care physician and oncologist.

**Table 1:** Key laboratory values during the diagnostic workup of splenomegaly and hypercalcemia EBV, Epstein-Barr virus; HIV, human immunodeficiency virus; PTH, parathyroid hormone; VCA, viral capsid antigen

Test Category	Result	Reference Range
Hematology		
White blood cell count, x10^3^/µL	7.3	4.0-10.0
Hemoglobin, g/dL	11.4	13.6-17.0
Platelet count, ×10^3^/µL	219	150-400
Metabolic and endocrine		
Calcium, mg/dL	14.9	8.6-10.5
Lactate dehydrogenase, U/L	406	125-243
Parathyroid hormone, pg/mL	<4	8.7-77.0
PTH-related protein, pg/mL	6	11-20
1,25-dihydroxyvitamin D, pg/mL	176	19.9-79.3
25-hydroxyvitamin D, ng/mL	20.4	20.0-50.0
Infectious and immunologic		
EBV VCA IgG, U/mL	600	0-17.9
EBV VCA IgM, U/mL	10	0-35.0

The patient's abdominal pain continued to worsen, and he returned to the ED four weeks later with acute pancreatitis. A CT of the abdomen demonstrated an edematous pancreas with surrounding inflammation. His serum lipase levels were elevated to 183 U/L (normal: < 78 U/L). The pancreatitis was attributed to severe hypercalcemia, with a calcium level of 13.9 mg/dL. Imaging also revealed increasing retroperitoneal lymphadenopathy and persistent massive splenomegaly, now measuring 20 cm. The patient was managed with supportive care and intravenous fluids, resulting in improvement of abdominal pain and normalization of calcium levels. Serum 1, 25-dihydroxyvitamin D levels were significantly elevated, a finding commonly associated with granulomatous diseases and lymphomas (Table 1). CT of the chest showed no evidence of thoracic lymphadenopathy. The patient also had a normal angiotensin-converting enzyme level and negative quantiferon testing, suggesting a low likelihood of granulomatous disease. Although a lymphoproliferative disorder was suspected to be based on worsening retroperitoneal lymphadenopathy and hypercalcemia, the patient declined tissue biopsy, given the recent EBV diagnosis, and opted for outpatient PET imaging. The patient remained hospitalized for three days and was discharged in stable condition with minimal abdominal pain.

One week later, an outpatient PET scan (Figure 2) was performed, which revealed extensive intensely hypermetabolic adenopathy involving the abdominal, retroperitoneal, mesenteric, perigastric, peripancreatic, and common iliac nodes, extending to the retro-crural region - findings highly suspicious for lymphoma. Within seven days, the patient presented again to the ED with abdominal pain, nausea, vomiting, dehydration, acute kidney injury (AKI) with a creatinine of 1.5 mg/dL (baseline: 0.9 mg/dL), hypercalcemia of 14.9 mg/dL, and new-onset ascites, requiring readmission. He was treated with intravenous fluids and intravenous calcitonin with resolution of the AKI and hypercalcemia. Approximately 2.2 L of ascitic fluid was removed via paracentesis, which was exudative but negative for malignant cells on pathology.

**Figure 2:**
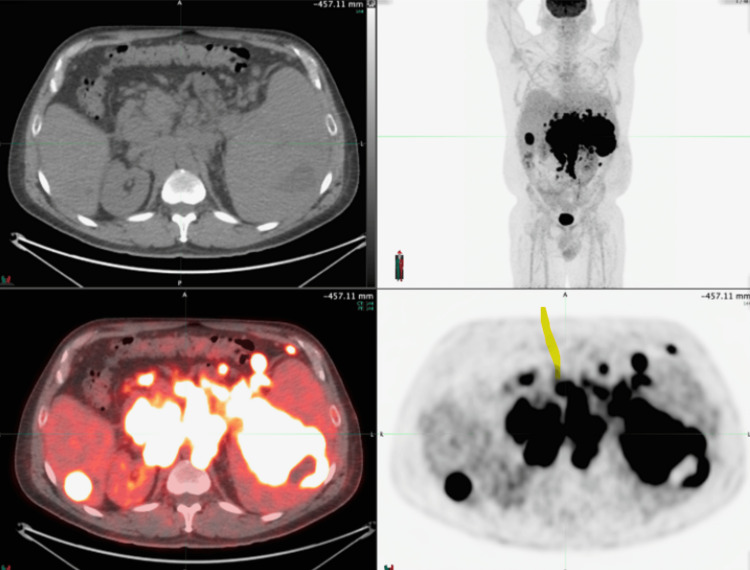
FDG-PET/CT showing hypermetabolic spleen, retroperitoneal lymph nodes, and liver lesion FDG, fluorodeoxyglucose

Given disease progression and high clinical suspicion for a lymphoproliferative disorder, the patient subsequently consented to a right axillary lymph node biopsy. An excisional lymph node biopsy was performed, and the patient was discharged home in stable condition to follow up with oncology as an outpatient.

Before he was able to attend outpatient oncology follow-up, the patient returned to the ED approximately two weeks later with abdominal distension and pain and was readmitted for recurrent ascites. Unexpectedly, the excisional lymph node biopsy showed no evidence of lymphoma or granuloma, and ascitic fluid cytology was also negative for malignant cells. As most diagnostic tests were unable to confirm the highly suspected lymphoproliferative disorder, a repeat CT imaging was performed. This identified a prominent 4.8 cm lesion in the right hepatic lobe, which was targeted for a biopsy (Figure 3). Ultrasound-guided biopsy of this lesion revealed CD45-positive, CD20-positive B cells (Figure 4) consistent with high-grade B-cell lymphoma. B-cell lymphomas are staged using the Lugano modification of the Ann Arbor staging system, and due to extra-lymphatic involvement (liver involvement in this case), this was classified as stage IV lymphoma. The patient was initiated on R-CHOP (rituximab, cyclophosphamide, doxorubicin, vincristine, and prednisone) chemotherapy and discharged in stable condition with outpatient oncology follow-up. His abdominal pain and distension improved.

**Figure 3:**
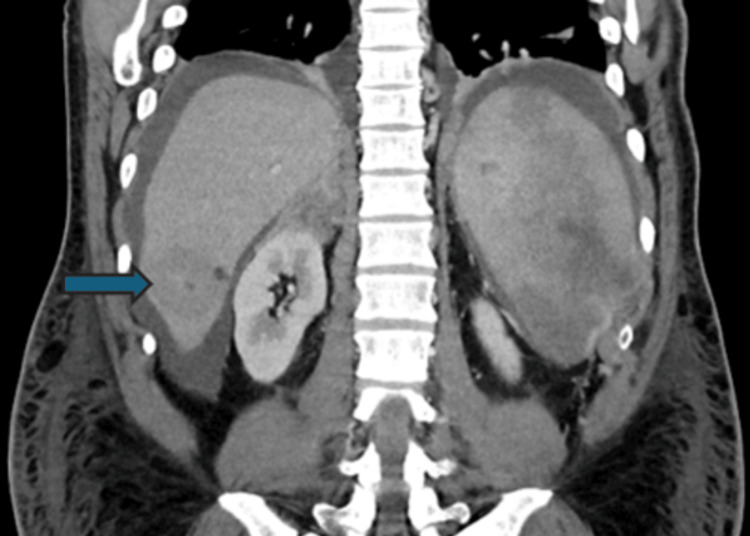
CT abdomen-pelvis showing a liver lesion, and the biopsy of this lesion was positive for lymphoma The blue arrow indicates the liver lesion.

**Figure 4:**
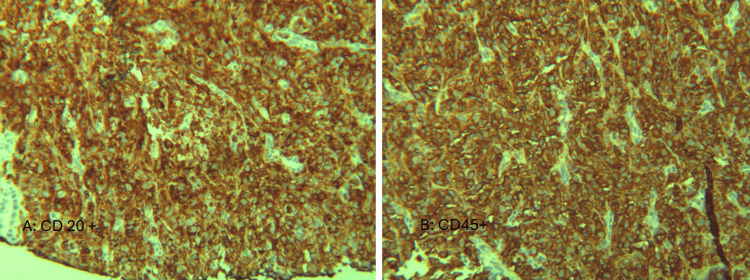
Histopathology of the liver lesion Immunohistochemical staining slide A showing CD20-positive cells and slide B showing CD45-positive cells.

Approximately two weeks later, the patient was readmitted for bilateral pleural effusions, ascites, and possible pneumonia, where he underwent thoracentesis and paracentesis and was treated with antibiotics. He was discharged in stable condition, and upon latest follow-up, he is awaiting his second cycle of chemotherapy. We have created a flow chart representing the patient's clinical course (Figure 5).

**Figure 5:**
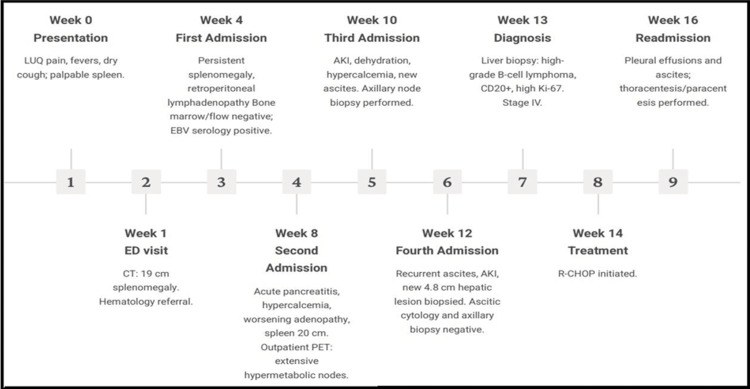
Flow chart illustrating the clinical course of our patient with significant events AKI, acute kidney injury; LUQ, left upper quadrant; R-CHOP, rituximab, cyclophosphamide, doxorubicin, vincristine, and prednisone

## Discussion

Splenomegaly encompasses a broad differential of diagnoses, ranging from self-limited viral illnesses to aggressive lymphoproliferative disorders. Infectious mononucleosis (IM) caused by EBV is among the most common causes. Its classic symptoms are fever, fatigue, pharyngitis, lymphadenopathy, and hepatosplenomegaly. The diagnosis of acute EBV infection is confirmed by the presence of anti-viral capsid antigen (VCA) IgM antibodies, which appear early in infection and typically disappear within four to six weeks. Anti-VCA IgG antibodies emerge during the acute phase, peak at two to four weeks after symptom onset, decline slightly, and then persist for life. The presence of anti-Epstein-Barr nuclear antigen (EBNA) IgG antibodies indicates that infection occurred at least 6-12 weeks prior and is suggestive of remote infection. In our patient, the serologic profile, negative VCA IgM with positive VCA IgG and positive EBNA IgG, was consistent with past rather than acute EBV infection [8].

Treatment of IM is supportive, with the majority of patients recovering within two to four weeks. In contrast, patients with lymphoma typically present with lymphadenopathy in the neck and abdomen. Approximately one-third of patients have B symptoms (fever, weight loss, night sweats), and 40% of patients present with extra nodal disease. Diagnosis is established through morphologic examination and immunophenotyping of excisional or incisional lymph node biopsy specimens.

Commonly reported diagnostic intervals in the literature include time from symptom onset to first presentation (9-91 days), first presentation to diagnosis (15-126 days), diagnosis to treatment initiation (1-42 days), and overall symptom onset to diagnosis (26-217 days). In our patient, the interval from first presentation to diagnosis was approximately 120 days [9]. This case highlights several significant diagnostic challenges in the evaluation of splenomegaly and lymphadenopathy.

Diagnostic Anchoring Bias From Positive EBV Serology

The presence of positive EBV serology contributed to a diagnostic anchoring bias early in the clinical course, as EBV-related reactive hepatosplenomegaly and lymphadenopathy are common. However, over 90% of the adult population harbors lifelong anti-VCA IgG antibodies, and rendering a positive IgG result alone is insufficient to attribute clinical findings to active EBV infection. Critically, EBV is itself implicated in the pathogenesis of several aggressive lymphomas, including DLBCL, Burkitt lymphoma, and Hodgkin lymphoma, through mechanisms involving viral latent membrane proteins and Epstein-Barr nuclear antigens that promote B-cell transformation and survival. Therefore, positive EBV serology does not exclude malignancy and may, in fact, be pathogenically linked to it.

Sampling Limitations of Initial Biopsies

The initial negative bone marrow biopsy and subsequent negative excisional lymph node biopsy in this case demonstrate the sampling limitations inherent in diagnosing lymphoproliferative disorders. Lymphoma involvement can be patchy, and peripheral lymph nodes may not always reflect disease at deeper sites. A large multicenter study from the French lymphopath network demonstrated that, while core needle biopsy provided a definitive lymphoma diagnosis in 92.3% of cases, excisional biopsy was superior at 98.1%, with lower diagnostic concordance observed for core needle biopsy, particularly for equivocal lesions [7]. Even though the reported diagnostic sensitivity of excisional biopsy is very high, the eventual diagnosis was established only after biopsy of a hepatic lesion, highlighting the importance of targeting the most metabolically active or clinically suspicious sites when initial lymph node biopsies are non-diagnostic. The National Comprehensive Cancer Network (NCCN) guidelines emphasize that a re-biopsy should be performed if the initial material is nondiagnostic, preferably from the most fluorodeoxyglucose (FDG)-avid, accessible site [10].

Hypercalcemia and Acute Pancreatitis as Clinical Red Flags

The development of severe hypercalcemia and acute pancreatitis served as critical clinical red flags (Table 2), suggesting aggressive malignancy rather than a benign viral process. Hypercalcemia in lymphoma can occur through several mechanisms, including humoral secretion of parathyroid hormone-related protein (PTHrP), local osteolytic bone resorption, and ectopic production of 1,25-dihydroxyvitamin D (calcitriol). In our patient, severe hypercalcemia was mediated by calcitriol overproduction, as confirmed by significantly elevated 1,25(OH)₂D levels. This mechanism is observed in sarcoidosis, tuberculosis, and lymphomas, where malignant cells or tumor-adjacent macrophages express the 1α-hydroxylase enzyme (CYP27B1), leading to unregulated conversion of 25-hydroxyvitamin D to calcitriol and subsequently increased intestinal calcium absorption and bone resorption [11]. A retrospective study of patients with non-Hodgkin lymphoma and hypercalcemia found that elevated calcitriol levels were associated with worse progression-free survival, potentially serving as a surrogate marker of more advanced disease [12]. Elevated serum LDH is a well-established marker of tumor burden and metabolic activity in lymphoma and is incorporated into multiple validated prognostic indices, including the International Prognostic Index (IPI) and the NCCN-IPI for DLBCL [13].

**Table 2:** Key clinical red flags that emerged during the patient's evaluation and prompted further investigation for a lymphoproliferative disorder EBV, Epstein-Barr virus; LDH, lactate dehydrogenase; CT, computed tomography; PET, positron emission tomography; TB tuberculosis

Red Flag	Clinical Significance
Deterioration after resolved EBV-related viral illness	Suggests alternative diagnosis
Severe hypercalcemia with elevated 1,25-dihydroxyvitamin D	Commonly seen in lymphomas, TB, sarcoidosis
Elevated LDH	Marker of tumor burden and metabolic activity
Progressive retroperitoneal lymphadenopathy on CT	Progressive disease process
Hypermetabolic lymphadenopathy on PET	High suspicion for malignancy

The discordance between the clinical presentation - progressive symptoms, hypercalcemia, extensive PET-avid adenopathy - and initial negative biopsies should prompt earlier repeat tissue sampling from alternative sites. This case underscores the principle that clinical suspicion should drive diagnostic persistence when initial workup does not adequately explain the clinical picture. As per NCCN guidelines, re-biopsy of the most FDG-avid accessible lesion is warranted when the initial material is nondiagnostic. Liver or spleen biopsy should be strongly considered when hepatosplenomegaly is present, and the evaluation for an underlying etiology has been otherwise unrevealing.

## Conclusions

This case illustrates the diagnostic complexity involved in differentiating EBV-related reactive processes from aggressive lymphoproliferative disorders. The presence of multiple negative biopsies, alongside positive EBV serology, contributed to diagnostic anchoring bias. This fostered a misleading sense of certainty that ultimately delayed appropriate diagnosis and treatment. Clinicians must maintain a high index of suspicion for malignancy, especially when clinical features are inconsistent with benign diagnoses. Progressive symptoms, hypercalcemia, and extensive PET-avid adenopathy are particularly concerning features. In cases of strong clinical suspicion, repeat tissue sampling from metabolically active sites is crucial when initial biopsies yield non-diagnostic results.
